# Modeling future wildlife habitat suitability: serious climate change impacts on the potential distribution of the Rock Ptarmigan *Lagopus muta japonica* in Japan’s northern Alps

**DOI:** 10.1186/s12898-019-0238-8

**Published:** 2019-07-10

**Authors:** Masanobu Hotta, Ikutaro Tsuyama, Katsuhiro Nakao, Masaaki Ozeki, Motoki Higa, Yuji Kominami, Takashi Hamada, Tetsuya Matsui, Masatsugu Yasuda, Nobuyuki Tanaka

**Affiliations:** 1 Natural Environment Division, Nagano Environmental Conservation Research Institute, 2054-120 Kitago, Nagano, 381-0075 Japan; 2 Hokkaido Research Center, Forestry and Forest Products Research Institute, 7 Hitsujigaoka, Toyohira, Sapporo, 062-8516 Japan; 3 Kansai Research Center, Forestry and Forest Products Research Institute, 68 Nagaikyutaroh, Momoyama, Fushimi, Kyoto, 612-0855 Japan; 4 Laboratory of Plant Ecology, Faculty of Science, Kochi University, 2-5-1 Akebono, Kochi, 780-8520 Japan; 5 Center for International Partnerships and Research on Climate Change, Forestry and Forest Products Research Institute, Matsunosato 1, Tsukuba-shi, Ibaraki-ken 305-8687 Japan; 6 Asia Air Survey Co., Ltd, Shinyuri 21 Building, 1-2-2 Manpukuji, Asao-ku, Kawasaki-shi, Kanagawa Prefecture 215-0004 Japan; 7 Department of International Agricultural Development, Tokyo University of Agriculture, 1-1-1 Sakuragaoka, Setagaya-ku, Tokyo, 156-8502 Japan

**Keywords:** Climate change impact, *Lagopus muta japonica*, Potential habitat, Rock Ptarmigan, Species distribution model

## Abstract

**Background:**

The Rock Ptarmigan *Lagopus muta japonica* lives in the alpine zones of central Japan, which is the southern limit of the global distribution for this species. This species is highly dependent on alpine habitats, which are considered vulnerable to rapid climate change. This study aimed to assess the impact of climate change on potential *L. muta japonica* habitat based on predicted changes to alpine vegetation, to identify population vulnerability under future climatic conditions for conservation planning. We developed species distribution models, which considered the structure of the alpine ecosystem by incorporating spatial hierarchy on specific environmental factors to assess the potential habitats for *L. muta japonica* under current and future climates. We used 24 general circulation models (GCMs) for 2081–2100 as future climate conditions.

**Results:**

The predicted potential habitat for *L. muta japonica* was similar to the actual distribution of the territories in the study area of Japan’s northern Alps (36.25–36.5°N, 137.5–137.7°E). Future potential habitat for *L. muta japonica* was projected to decrease to 0.4% of the current potential habitat in the median of occurrence probabilities under 24 GCMs, due to a decrease in alpine vegetation communities. Some potential habitats in the central and northwestern part of the study area were predicted to be sustained in the future, depending on the GCMs.

**Conclusions:**

Our model results predicted that the potential habitats for *L. muta japonica* in Japan’s northern Alps, which provides core habitat for this subspecies, would be vulnerable by 2081–2100. Small sustainable habitats may serve as refugia, facilitating the survival of *L. muta japonica* populations under future climatic conditions. Impact assessment studies of the effect of climate change on *L. muta japonica* habitats at a nationwide scale are urgently required to establish effective conservation planning for this species, which includes identifying candidate areas for assisted migration as an adaptive strategy.

**Electronic supplementary material:**

The online version of this article (10.1186/s12898-019-0238-8) contains supplementary material, which is available to authorized users.

## Background

Recent climatic warming has strongly influenced a wide range of species and communities in terrestrial ecosystems, with responses that include consistent poleward and elevational range shifts for flora and fauna [1]. Engler et al. [2] predicted that 36–55% of alpine plant species, 31–51% of subalpine plant species, and 19–46% of montane plant species in Europe will lose more than 80% of their suitable habitat by 2070–2100. Sekercioglu et al. [3] predicted that approximately 30% of land birds will be at worldwide extinction risk by 2100. A study of the northern and southern range limits of British birds showed that the northern range boundaries of 59 species will move 18.9 km northwards on average over a 20-year period [4].

The average global temperature has increased by 0.74 °C over the last century, and it is projected to increase by a further 1.8–4.0 °C by 2090–2099 [1]. If a significant cut in global greenhouse gas emissions is achieved, temperature rise may stabilize at approximately 2 °C; however, even with this limit, a certain amount of damage is unavoidable [1]. It is thus necessary to assess the impact of climate change on species distributions to determine the extent of potential ecological risk, and to design appropriate conservation strategies for adaptive management.

Reducing the vulnerability of species and ecosystems to climate change is an increasingly important conservation objective [5]. Identifying areas likely to become important for vulnerable populations is necessary to assist their adaptation to climate change. Artificial climate change experiments, such as those involving rainfall and CO_2_ manipulation, have been widely used to elucidate how species will respond to future climate change, particularly for plant species and communities [6]. Several ecological phenomena, including phenological change and advancement of spring events, which are considered to be related to rising temperature, have been detected in recent years [7]. However, for animal species, it is difficult to detect signs of climate change impacts using artificial climate change experiments because of their higher mobility. Therefore, quantitative assessments that identify current and future areas of particular conservation concern for populations are required to facilitate adaptive management practices. Species distribution models (SDMs) have been recognized as a useful tool for assessing the impact of environmental change on the distribution of organisms, and for selecting natural ecosystems important for preservation and management [8, 9]. Although several advantages of using SDMs projections in conservation planning have been acknowledged [10], the projections include uncertainties caused by such as variation of climate models (i.e. GCMs) [11]. The inclusion and reduction of uncertainties in conservation planning is, therefore, important to increase the reliability of spatial conservation planning [12].

Montane and alpine species are expected to be considerably influenced by global warming (e.g., the American pika [13], the White-tailed Ptarmigan [14], the Black Grouse [15]). In particular, alpine plants and animals in Japan are considered to be vulnerable to climate change, because they already inhabit mountain tops, and consequently cannot shift to higher elevation refuges in response to global warming. The Rock Ptarmigan *Lagopus muta*, which has low fecundity, high survival, a long generation time, and slow population recovery [16], is adapted to arctic and alpine environments, with some southern populations being isolated in alpine habitats as relicts of the glacial epoch [17]. In the Swiss Alps, Revermann et al. [18] predicted that up to two-thirds of potential *L. muta helvetica* habitat will be lost by 2070 and showed that potential habitat would shift toward mountaintops by using several statistical modeling approaches, including species distribution models. Consistent with this prediction, in some regions of the Swiss Alps, volunteer observers have increasingly recorded Ptarmigans at higher elevations over the last three decades [19]. In Europe, *L. muta* populations are likely to decline under warming scenarios, with a projected 54% decline in suitable climate space from the present to the 2080s [20].

According to the IUCN Red List, the worldwide status of *L. muta* is of least concern [21]. However, in Japan, populations of the subspecies (*L. muta japonica*) have already become extinct in some mountain areas [22]. In the 1980s, the population of *L. muta japonica* was estimated at 3000 birds. In the northern part of Japan’s Southern Alps, the number of breeding pairs declined by ~ 50% from 1981 to 2004 [22, 23]. In addition, there is concern that the grazing impacts of increasing Sika deer *Cervus nippon* populations [24, 25], along with an increase in montane terrestrial predators, will negatively affect the flora and fauna of the alpine zone. As a consequence, the ranking of *L. muta japonica* was raised from vulnerable to endangered in the Japanese Red List revised in August 2012 [26]. On the basis of the law for the conservation of endangered wild fauna and flora species, the Ministry of the Environment introduced a program for the conservation of *L. muta japonica* in October 2012. In central Japan, the subspecies *L. muta japonica* inhabits the mountain alpine zone, which represents the southernmost limit of the global distribution of this species [27]. *L. muta japonica* uses the Japanese stone pine (*Pinus pumila*), which is a dominant alpine zone shrub species, as nesting sites and shelters from predators. *L. muta japonica* mainly feeds in alpine fellfields and snowbed grasslands [22, 28]. Consequently, this species is highly dependent on alpine habitats, which are considered vulnerable to rapid climate change [29]. Therefore, *L. muta japonica* is hypothesized to be influenced by changes to alpine vegetation due to global warming. Of particular concern in this regard is the prediction by Nakamura that most of the available habitat for *L. muta japonica* would disappear if the annual mean temperature rises by 3 °C [22].

In the present study, to assess the vulnerability of *L. muta japonica* populations and identify sites for conservation planning under future climate change, changes in the potential habitat for *L. muta japonica* were predicted using an SDM, which describes how the alpine ecosystems interact with 24 future climate simulations. The results are expected to identify sustainable and vulnerable habitats of *L. muta japonica* in the study area, which could be integrated into the conservation planning for the species under future climate change.

## Methods

### Study area

**Fig. 1:**
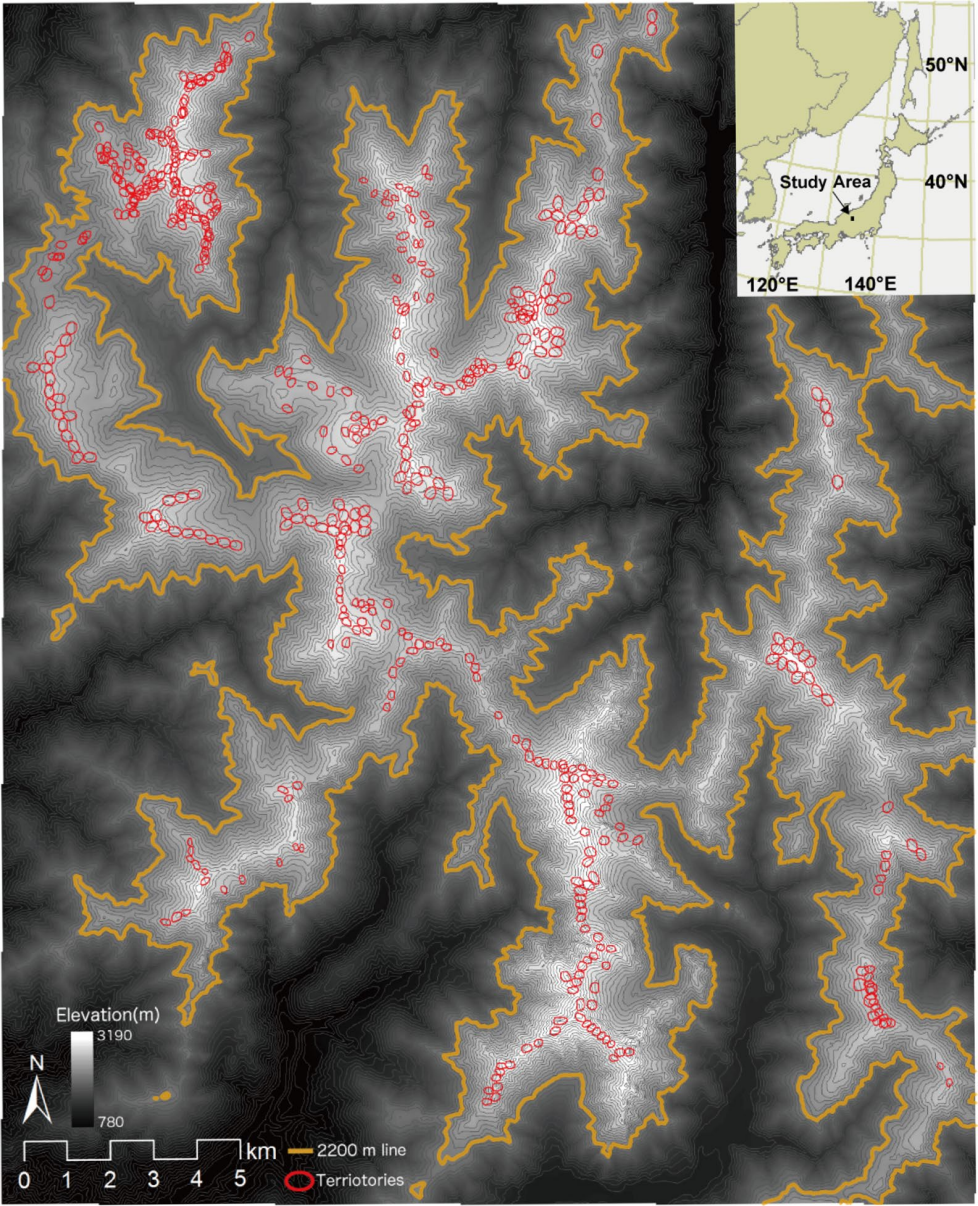
A map of the study area (20 km × 30 km) and the distribution of the Rock Ptarmigan (*Lagopus muta japonica*) territories in the southern part of Japan’s northern Alps (36.25–36.5°N, 137.5–137.7°E). Gray lines show contours at intervals of 50 m. Thick yellow lines indicate the altitude of 2200 m, which is the lower limit of *L. muta japonica* habitat. Territories of *L. muta japonica* are shown in the red ellipses

The study site was a 20 km × 30 km area in the southern part of Japan’s northern Alps (36.25–36.5°N, 137.5–137.7°E), of which the highest peak is 3190 m (Fig. 1). The study area supports approximately one-third of the current *L. muta japonica* populations, and so represents a major component of *L. muta japonica* habitats. The area has deep snow in winter, due to the influence of continental polar air from Siberia and moisture from the warm currents of the Sea of Japan. The alpine vegetation, which occurs above the forest limit at approximately 2200 m above sea level, consists of Japanese stone pine (*P. pumila*) scrub, snowbed grasslands (including *Phyllodoce aleutica*, *Sieversia pentapetala*, *Fritillaria camschatcensis*, and *Primula cuneifolia* var. *hakusanensis*), and alpine fellfields (alpine heathland and wind-exposed grassland) dominated by dwarf shrubs (including *Arcterica nana*, *Empetrum nigrum* var. *japonicum*, *Vaccinium uliginosum*, and Poaceae grasses).

### Data construction

#### *L. muta japonica* data

In the study area, the *L. muta japonica* population size was estimated at 352–376 territories and 860–920 birds, based on the results of intermittent surveys from 1971 to 2010 (Additional file 1: Appendix S1). By way of comparison, the total number of *L. muta japonica* in the 1980s was approximately 3000 birds [22]. In this analysis, we used the distribution of territories as occurrence data of *L. muta japonica*. In Japan, *L. muta japonica* lives in the alpine zone of the mountains above 2200 m. The mean territory size of cocks (males) during the breeding season ranges from 0.015 to 0.072 km^2^ [22, 30]. On the basis of this information, we defined the grid cell size for projection of the potential habitat as 300 m (0.09 km^2^). Grid cells that did not overlap with the territories were defined as absence.

#### Vegetation data

We used a digitized vegetation map provided by the Ministry of the Environment of Japan (http://www.biodic.go.jp/trialSystem/EN/info/vg67.html, accessed in September 2012, Additional file 2: Figure S1). The data were constructed based on the 6th and 7th national surveys on the natural environment (2001 and 2003), which had a scale of 1:25,000. We extracted natural alpine vegetation in three communities: Japanese stone pine (*P. pumila*), snowbed grassland, and alpine fellfield. We divided the study area into 60,000 grid cells, based on the stipulated 300-m grid cell size. Area fractions for each community type in the Rock Ptarmigan territories and 300-m grid cells were calculated.

#### Climatic data

Climatic data for grid cells, with a resolution of 30″N × 45″E (i.e. ~ 1 km resolution, ca. 1 km^2^), obtained from the Japan Meteorological Agency [31], were used to model the current distribution of alpine vegetation. The dataset is calculated from meteorological temperature observations collected between 1953 and 1982 and precipitation data collected between 1953 and 1976. Four climatic factors considered important for forming alpine vegetation were used as explanatory variables in the species distribution model: (1) the warmth index, which is defined as the annual sum of positive differences between monthly mean temperature and + 5 °C [32] and provides a measure of effective heat quantity required for plant growth; (2) monthly mean daily minimum temperature in the coldest month (minimum temperature), which is a measure of extreme cold; (3) summer (May–September) precipitation, which provides a measure of water supply during the growing season; and (4) maximum snowwater equivalent (maximum snowwater), which provides a measure of snow accumulation. Maximum snowwater was calculated by aggregating daily changes in snowfall, rainfall, and meltwater (degree-day method) based on the daily climatic dataset supplied from the Automated Meteorological Data Acquisition System. Snow disappearance dates were derived from 10-day composites of Satellite data and climatic data for grid cells [33, 34].

#### Future climate change scenarios

To assess the impact of climate change by incorporating the uncertainties related to general circulation models (GCMs), we applied 24 GCMs from the World Climate Research Programme (WCRP) Coupled Model Intercomparison Project phase 3 (CMIP3) multi-model dataset [35] as future climates (Additional file 3: Table S1). The climate change scenarios were generated based on scenario A1B of the Intergovernmental Panel on Climate Change (IPCC) Special Report on Emission Scenarios, which assumed rapid economic growth, the rapid spread of new and efficient technologies, and a balanced emphasis on all energy sources [36]. The A1B scenario is most commonly cited as a high emission scenario in the Fourth Assessment Report of IPCC [1]. We selected the A1B scenario as a possible future because we considered that we should assess the worst case first for the purpose of conservation. The A1B scenario is similar to between Representative Concentration Pathways 6.0 and 8.5 in the later climate scenarios CMIP5 in the Fifth Assessment Report of IPCC, which is second and highest emission scenarios [37]. We only used the data for the period 2081–2100 because the fractional uncertainty for natural factors of internal variability (such as the El Niño Southern Oscillation, which occurs every few years or decades) is larger in shorter-term climate scenarios. Such internal variability is a noise for global warming prediction, which would emphasize or cancel the effects of greenhouse gases [38]. The climate of the twentieth century experiment (20c3m) was used as baseline for current climate. We calculated differences in the means and minimum daily temperatures, and ratios of precipitation between 1961 and 1980 of 20c3m and 2081 and 2100 of the A1B scenario. These differences and ratios were spatially interpolated from original spatial scales (approximately 125 km to 444 km in latitude) to 1-km grid cells using a simple linear interpolation method, and then applied to the current climatic data (i.e. climatic data for grid cells) and used as future climatic data. The four climatic variables in each grid cell were calculated using the future climate scenarios in the same manner as the calculations for current climate. When comparing the current climate to the future climate scenarios within the study area, warmth index and minimum temperature increased in all scenarios, summer precipitation increased in all but four scenarios, and maximum snowwater decreased in all scenarios (Additional file 3: Table S1).

#### Topographic data

We constructed topographic data based on a 10-m-resolution digital elevation model that was supplied by the Geospatial Information Authority of Japan by using the System for Automated Geoscientific Analyses (SAGA, URL: http://www.saga-gis.org/en/index.html). The topographic data were constructed at 100-m resolution, which was assumed appropriate for estimating the fraction of the areas for alpine vegetation. Five topographic factors, which are thought to be important for plant growth, were calculated, namely, inclination, slope direction, curvature, topographic wetness index (TWI) [39], and distance from the ridgeline.

Variation in the cover and height of *P. pumila* is closely related to the degree of wind exposure and snow depth in high mountains during winter, together with microtopographic change. The height of *P. pumila* decreases gradually as the degree of wind exposure increases [40]. The degree of wind exposure is higher closer to the ridgeline on the wind-blown slope side in winter [41]. Thus, distance from the ridgeline was measured as the topographic factor that is considered to affect the height of the *P. pumila* community.

### Model construction

#### Species distribution models

**Fig. 2:**
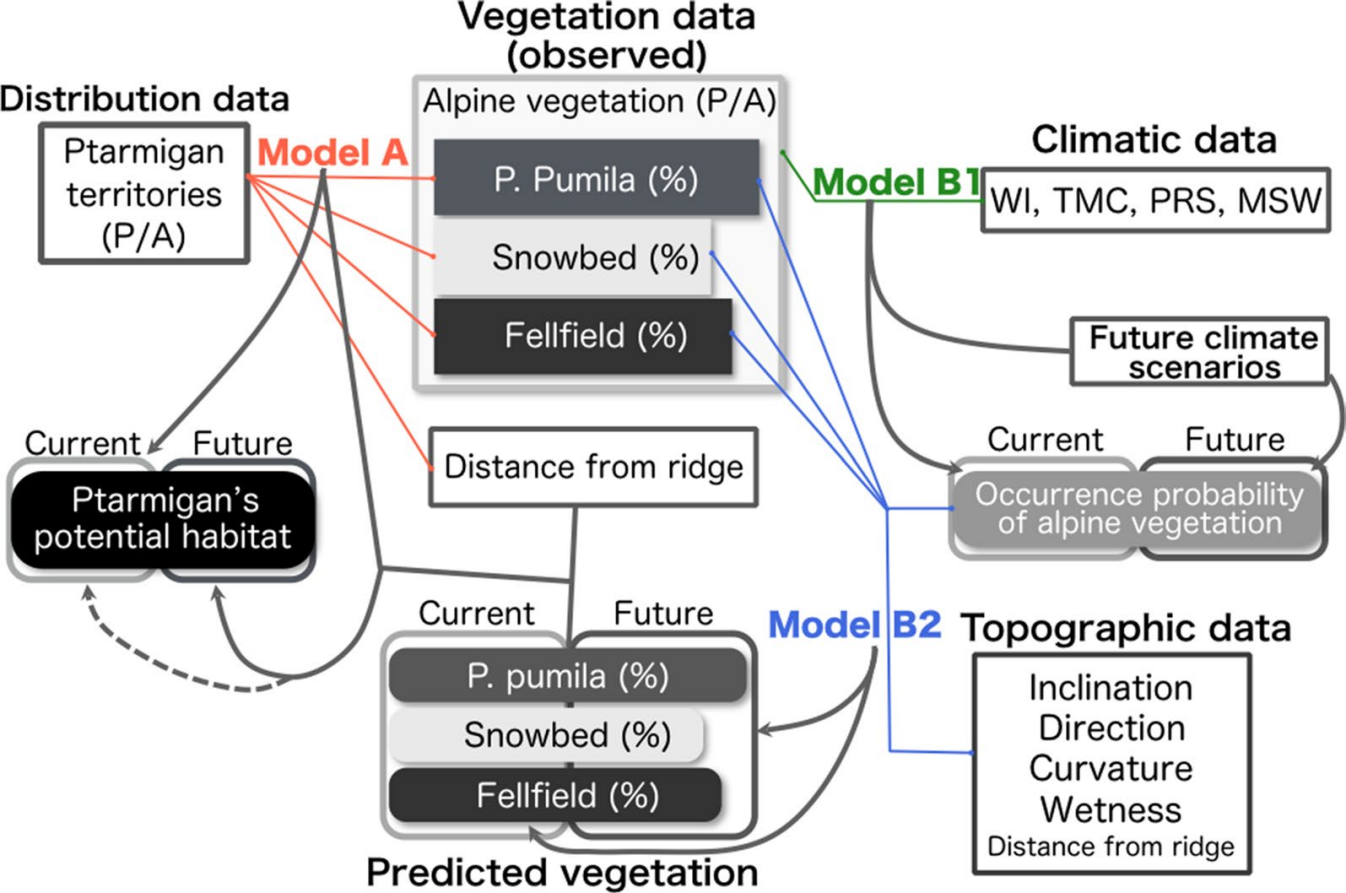
Model scheme for assessing the impact of climate change on the distribution of *Lagopus muta japonica* in the alpine zones of central Japan. Abbreviations in this scheme are as follows: WI (warmth index), Warmth Index; TMC (minimum temperature), minimum temperature of the coldest month; PRS (summer precipitation), precipitation of the coldest month; MSW (maximum snowwater), maximum snowwater equivalent (see “Methods” for details)

To assess the impact of climate change on the distribution of *L. muta japonica* in the alpine zones of central Japan, we developed a species distribution model that included three sub-models (Fig. 2). The model was constructed based on the hypotheses that the functional relationships of *L. muta japonica* depend highly on the alpine plant communities in terms of quantity and quality, which depend on climate at macro-scales and topography at small scales. The model was constructed in two main parts. The first part of the model aimed to predict potential habitat for *L. muta japonica* based on the area fractions of three alpine vegetation communities and distance from the ridge (sub-model A). The second part of the model aimed to predict the area fractions of alpine vegetation communities based on climatic and topographic variables (sub-model B1 and B2). Potential habitats for *L. muta japonica* under future climate change scenarios were projected by applying the future area fractions of alpine vegetation communities, which were predicted from sub-model B2, on sub-model A. All of the models (sub-model A, B1 and B2) were developed by using R2.15.2 [42]. Detailed explanations of each model are provided in the following sections.

#### Sub-model A for predicting the potential habitat of *L. muta japonica*

A generalized additive model (GAM) [43] was used to predict the potential habitat for *L. muta japonica*. We used the package “mgcv” to construct a GAM in R. Data on the presence and absence of territories for *L. muta japonica* were used as a response variable. The area fractions of three alpine vegetation communities in the territories and 300-m grid cells of absent areas were used as explanatory variables, together with distance from the ridge. The three communities used were the *P. pumila*, snowbed grassland, and alpine fellfield communities. The number of surveys was included as an offset term, to incorporate the effect of survey effort. The area fraction of the *P. pumila* community was used as a combination term with distance from the ridge, based on the thin plate smoothing spline algorithm (see Additional file 4: Appendix S2). The relative importance of the variables (IOV) was calculated based on the Akaike weight for all of the models, which was developed by all combinations of explanatory variables [44].

We validated the accuracy of the model by calculating the area under the curve (AUC) of the receiver operating characteristic (ROC) analysis [45] based on predicted presence/absence data and the validation data, which was bootstrapped from all of the training data with 100 repetitions. We chose using whole data for modeling and bootstrapped data for validation because we considered development of good model more important than the robustness of validation (although validation results could be slightly overestimated). The AUC ranges from 0.5 for models with no discrimination ability to 1.0 for models with perfect discrimination [45]. In addition, we calculated model sensitivity and specificity (the proportions of presence and absence data correctly predicted) based on the predicted and actual presence/absence data of *L. muta japonica* territories. We defined a threshold value by assigning a cutoff sensitivity (95%), as recommended by Pearson et al. [46]. The threshold was defined based on model predictions for the entire training data set. We defined areas with probabilities greater than, or equal to, the threshold probability as potential habitat, and areas with probabilities lower than the threshold value as non-habitat.

#### Sub-models B1 and B2 for predicting the area fractions of alpine vegetation communities

We developed two sub-models to predict the area fractions of alpine vegetation communities (B1 and B2). In general, plant species distributions are assumed to be controlled by climate at large scales and by non-climatic factors at small scales, including topography, soil conditions, and biotic interactions [47]. In the present study, we assumed that climatic conditions control the occurrence of alpine vegetation zones at macro-scales, whereas topographic conditions with climatic suitability control the area fractions of alpine vegetation communities at micro-scales. On the basis of this assumption, we constructed two sub-models by using different explanatory variables at different spatial scales for each sub-model as follows.

We used a GAM to predict the occurrence of the alpine vegetation zone (sub-model B1), which contained the *P. pumila* community, snowbed grassland community, and alpine fellfield community, at 100-m grid cells (ca. 0.01 km^2^). Four climatic variables—warmth index [32], minimum temperature, summer precipitation, and maximum snowwater—at 1-km resolution were used as explanatory variables. Warmth index was used as an interaction term with minimum temperature based on the thin plate smoothing spline algorithm [43]. Model accuracy was evaluated by using the AUC together with sensitivity and specificity. Current and future occurrence probabilities of the alpine vegetation zone were predicted by applying the GAM on the current climate and 24 future climate scenarios.

We used random forests (RF) [48] to predict area fractions of alpine vegetation communities (sub-model B2). The package “randomForest” was used for constructing an RF in R. The RF is a model that is based on the machine learning method and has a powerful and high generalizing capability. The model was developed based on the ensemble of numerous classification tree models, by using sub-sampled data from the training data. Area fractions of alpine vegetation communities in each grid cell were used as response variables. As explanatory variables, we used five topographic variables (slope, aspect, curvature, TWI, and distance from the ridgeline) and the occurrence probability of the alpine vegetation zone calculated by sub-model B1. The occurrence probability of the alpine vegetation zone was used as an indicator of climatic suitability for each alpine vegetation community. Correlations between actual and predicted values of area fractions were verified by using Spearman’s rank sum test. The importance of each variable was identified based on increased mean square errors (increased MSE). Increased MSE measures the effect on the predictive power when the value of a specific original variable is randomly permuted [49].

## Results

### Habitat and current potential habitat predictions for *L. muta japonica*

Sub-model A, which predicted the occurrence of *L. muta japonica* from the area fractions of three alpine vegetation communities and distance from the ridgeline, showed reasonably accurate predictability (AUC: 0.97, sensitivity: 0.94, specificity: 0.93, threshold probability for potential habitat: 0.02). The IOV of the explanatory variables ranged from 0.99 to 1.00, which showed that all of the explanatory variables were important to the same extent for the occurrence of *L. muta japonica* (see Additional file 5: Table S2). Response curves of the model (Additional file 6: Figure S2), which show positive or negative effects on the occurrence probability in the model at plus or minus values of linear predictor, showed that the occurrence probability was higher in areas with high area fractions of the *P. pumila* community and close to the ridgeline. The fellfield community was positively affected when its area fraction was higher than 14.6%, but was negatively affected when the fraction was less than this value. The snowbed grassland community was positively affected in most of the area fractions, but was negatively affected when the fraction was less than 0.7%.

**Fig. 3:**
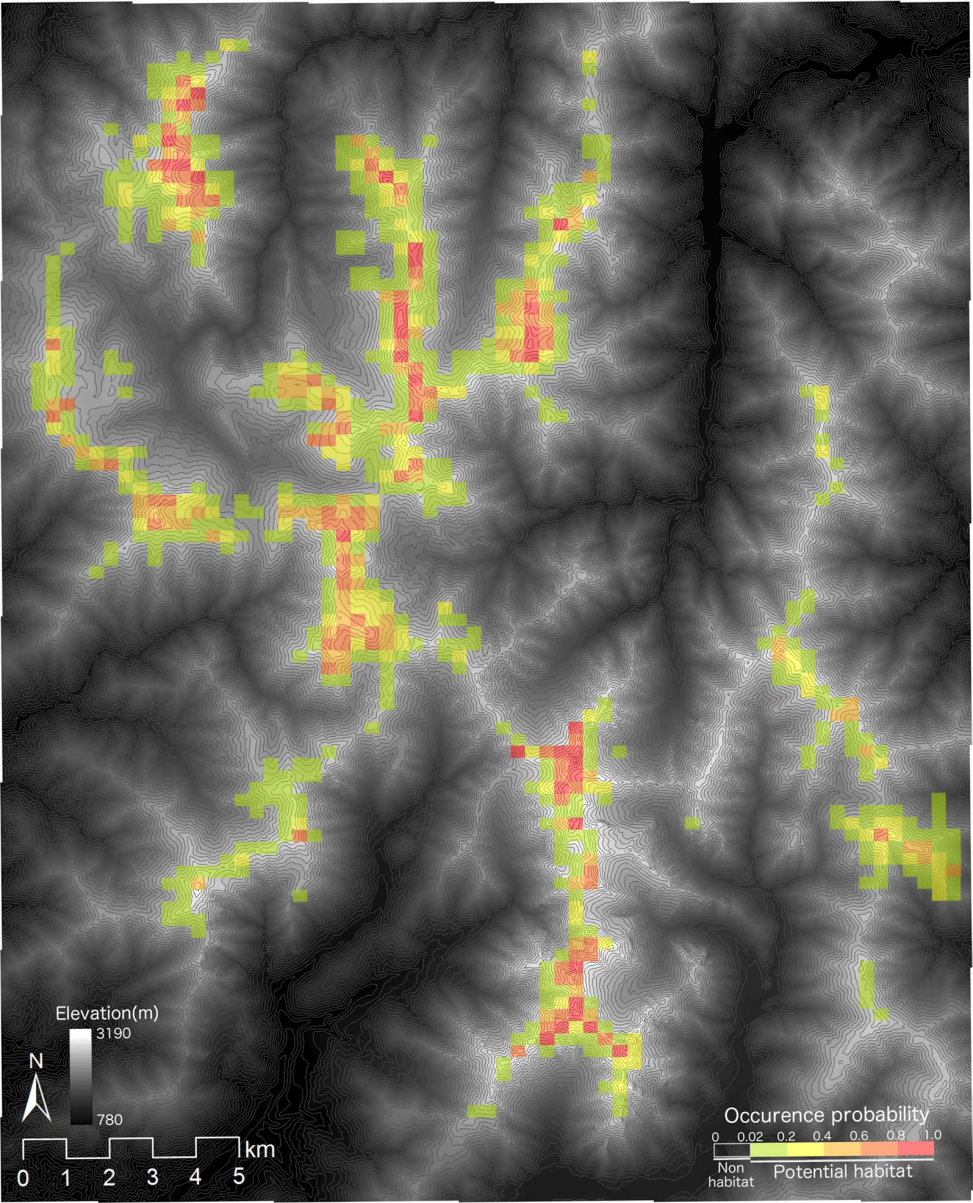
Current potential habitat for *Lagopus muta japonica* predicted by sub-model A. The areas with colors show potential habitat for *L. muta japonica.* The warmer the color, the higher the occurrence probability. Gray lines show contours at intervals of 50 m

The predicted potential habitat for *L. muta japonica* was comparatively similar to the actual distribution of the territories in the study area (Figs. 1 and 3). However, empty habitat (i.e. areas with suitable environmental conditions but no current occurrence of the species) was found in some areas (Fig. 3).

### Habitats for alpine vegetation

The indicators of model accuracy for sub-model B1 showed that the model was reasonably accurate (AUC: 0.96 ± 0.001, sensitivity: 0.95, specificity: 0.78, threshold probability for potential habitat: 0.08). The IOV of all the explanatory variables was almost 1.00, which showed that all of the climatic variables were important for the occurrence of the alpine vegetation zone (see Additional file 7: Table S3). Response curves of the GAM for sub-model B1 showed that higher warmth index strongly and negatively affected the occurrence of the alpine vegetation zone (Additional file 8: Figure S3). Summer precipitation higher than ca. 1500 mm had a positive effect on the alpine vegetation zone, whereas summer precipitation lower than ca. 1500 mm had a negative effect. Maximum snowwater higher than ca. 530 mm had a positive effect on the alpine vegetation zone, whereas maximum snowwater lower than ca. 530 mm had a negative effect.

Reasonably accurate models were developed to predict the area fractions of alpine vegetation communities by using topographic variables with the occurrence probability of the alpine vegetation zone from sub-model B1 (Spearman’s rank sum test, *P. pumila* community: *rho *= 0.90, *p* < 0.001, snowbed grassland community: *rho* = 0.44, *p* < 0.001, fellfield community: *rho *= 0.63, *p* < 0.001).

Increased MSE (see “Methods” for the definition) showed the importance of the variables for the three alpine vegetation communities (Additional file 9: Figure S4). Distance from the ridge was the most important factor for the area fraction of the *P. pumila* community, followed by occurrence probability of the alpine vegetation zone and slope direction. Occurrence probability of the alpine vegetation zone was the most important factor for the snowbed grassland community, followed by inclination, slope direction, and curvature. The occurrence probability of the alpine vegetation zone was the most important factor for the fellfield community, followed by TWI, inclination, curvature, and slope direction.

### Predicted potential habitat for *L. muta japonica* from the estimated area fractions of the alpine vegetation communities under current and future climates

We obtained reasonably accuracy in predicting the potential habitat for *L. muta japonica* by applying sub-model A to the estimated area fractions of the alpine vegetation communities from sub-model B2 (AUC: 0.92 ± 0.004, sensitivity: 0.95, specificity: 0.88).

**Fig. 4:**
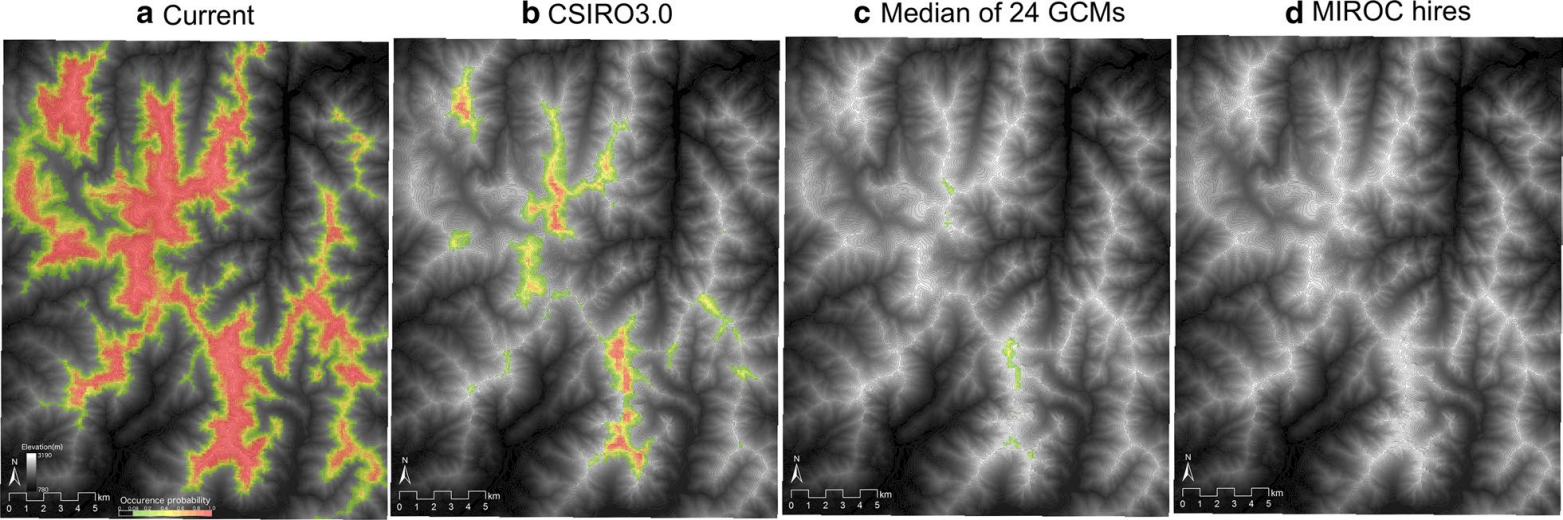
Potential habitats for the alpine vegetation zone under the current climate (**a**) and future climate scenarios for the period 2081–2100 (**b**–**d**). The areas with colors show potential habitat for the alpine vegetation zone. The warmer the color, the higher the occurrence probability. **b** The potential habitat under the scenario of minimum increase in temperature (CSIRO3.0). **c** The potential habitat derived from the median of the occurrence probabilities under 24 future climate scenarios. **d** The potential habitat under the scenario of maximum increase in temperature (MIROC hires). Gray lines show contours at intervals of 50 m

**Fig. 5:**
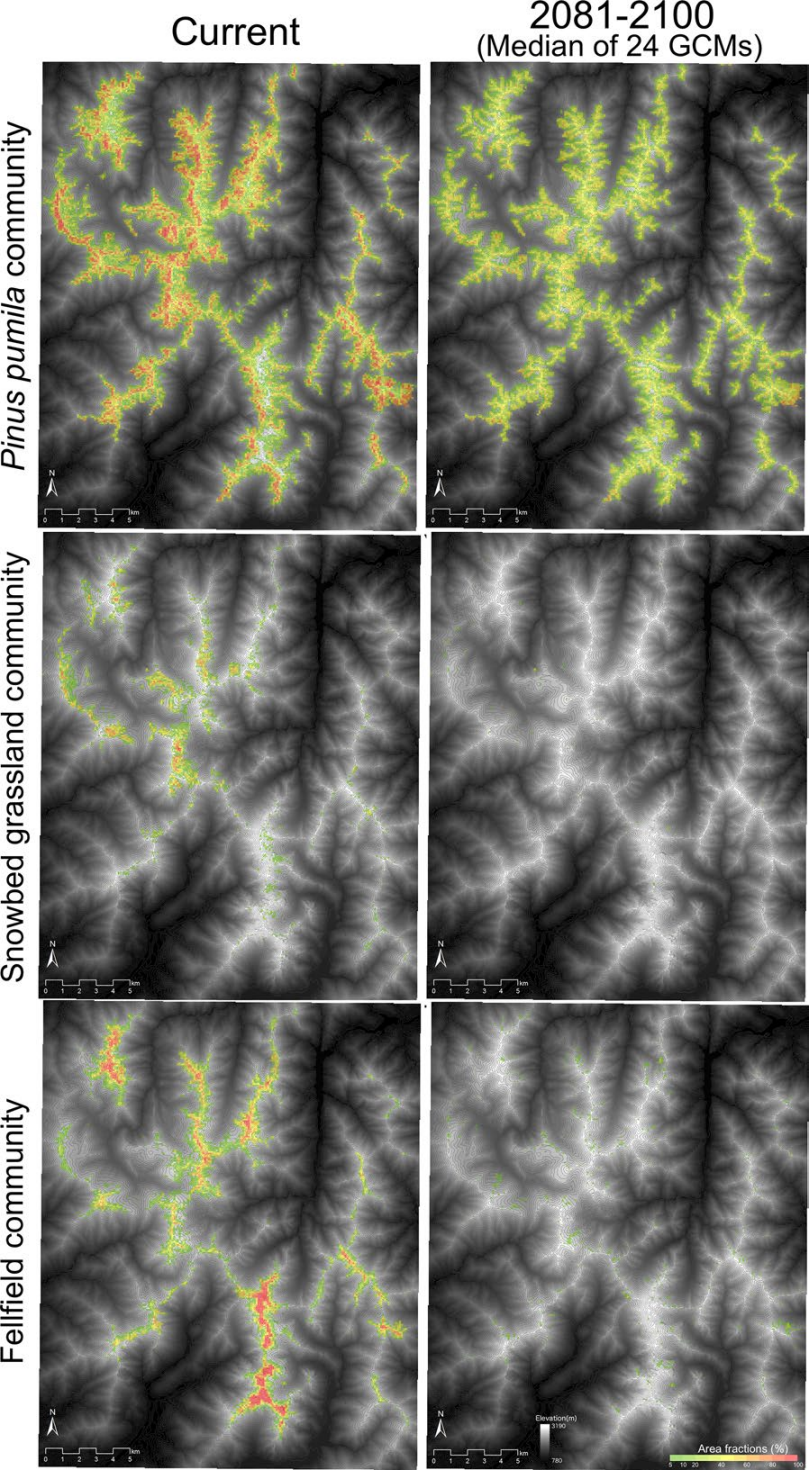
Current and future area fractions of the three alpine plant communities calculated from sub-model B2. The future area fraction is represented by the median value of area fractions under 24 future climate scenarios. Gray lines show contours at intervals of 50 m

**Fig. 6:**
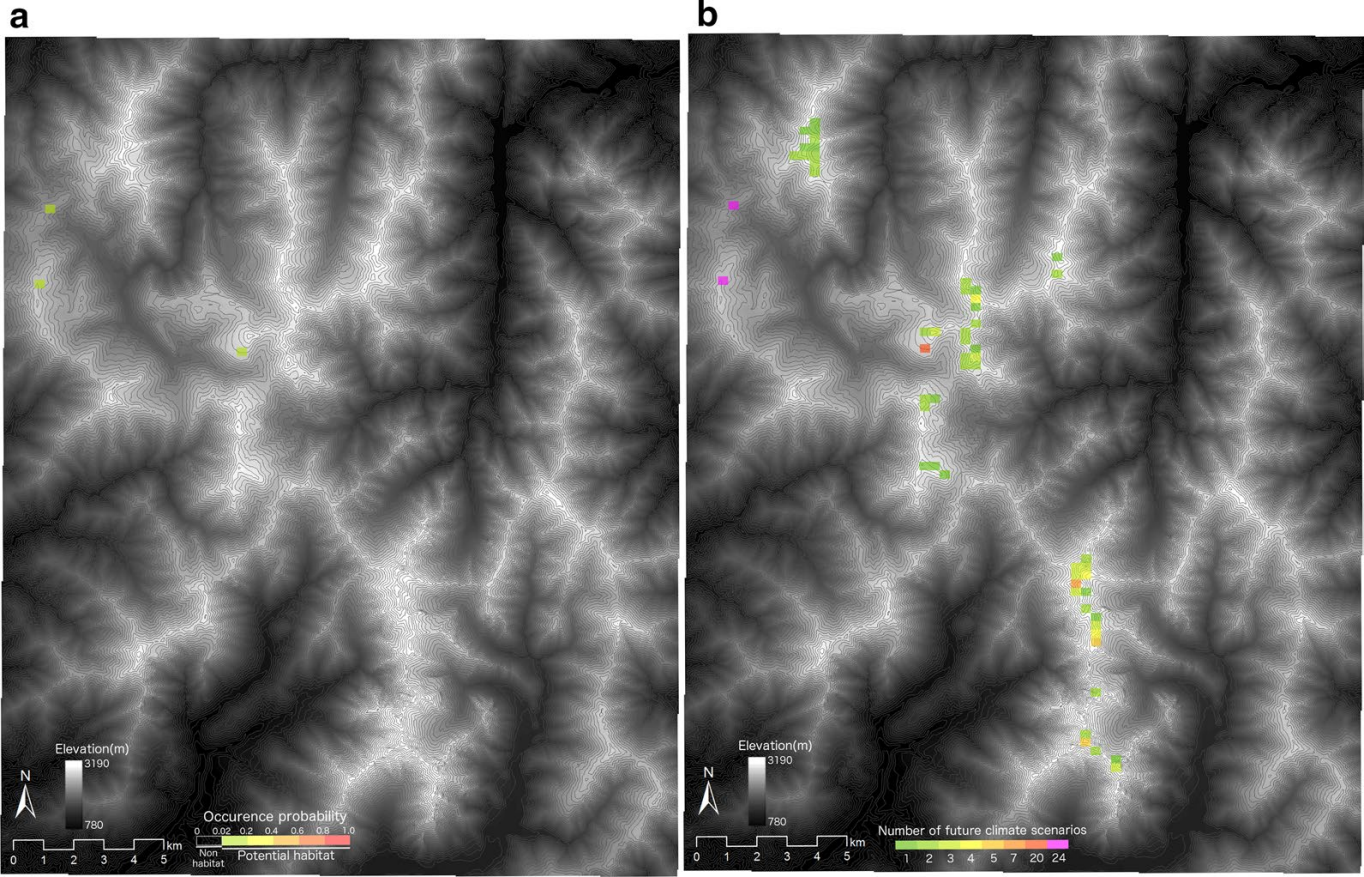
**a** Future potential habitat for *Lagopus muta japonica* represented by the median value of occurrence probabilities under 24 future climate scenarios, **b** numbers of climate scenarios under which potential habitat of *L. muta japonica* was predicted to be sustained. Gray lines show contours at intervals of 50 m

Potential habitat for the alpine vegetation zone was predicted to decrease from current (ca. 2464 km^2^) to future (ca. 18 km^2^, representing 0.7% of current in the median of 24 GCMs) (Fig. 4). Area fractions of all the three alpine vegetation communities were predicted to decrease under future climates (Fig. 5). Future potential habitat for *L. muta japonica* was projected to decrease to just three grid cells (i.e. 0.4% of current potential habitat) in the median of occurrence probabilities under 24 GCMs, due to a decrease in the area fractions of the alpine vegetation communities (Figs. 5 and 6a). Most potential habitat for *L. muta japonica* was predicted to be non-habitat under any future climate scenario, whereas some potential habitat in the central and north-western part of the study area was predicted to be sustained, depending on the GCM (Fig. 6b). Environments containing sustainable habitat for *L. muta japonica* included a climate with cooler temperatures, higher snowfall, and lower summer precipitation, and were located closer to ridges on the leeward (i.e. eastern) side and had a gentler slope in topography.

## Discussion

### Predicted potential habitat for *L. muta japonica* at the territory scale

This study predicted the potential habitats for *L. muta japonica* under current and future climates at the territory scale (i.e. 300 m × 300 m, 0.09 km^2^) by developing a model that considered the structure of the alpine ecosystem through incorporating spatial hierarchy on various environmental factors. Predicted current potential habitat was consistent with the current observed distribution of *L. muta japonica* territories, indicating that alpine vegetation communities, such as the *P. pumila*, snowbed grassland, and fellfield communities, are essential for *L. muta japonica*. Our findings are quantitatively consistent with previous studies on the ecology of *L. muta japonica* in Japan [49–51].

Incorporating both large scale (i.e. climatic) and smaller scale factors, including biological interactions, microtopography, and microclimate, is important for realistic predictions of species distributions, which is essential for practical spatial conservation planning [46, 52]. Previous studies that have predicted the potential habitat for *L. muta japonica* in Europe were conducted at the macro-scale (100 km^2^) and meso-scale (1 km^2^), mainly taking climatic factors into consideration [18, 20]. Our study is the first to incorporate the effects of environmental factors at a micro-scale (i.e. the territory scale) to predict the potential habitat of *L. muta japonica*.

### Changes in alpine vegetation habitat under future climates

We predicted changes in the area fractions of alpine plant communities under future climate by incorporating both climatic factors at the macro-scale and topographic factors at a smaller scale. Our study clarified that climatic suitability for alpine vegetation is the most important factor that determines the area fractions of alpine plant communities, whereas the importance of topographic factors differed in each community type.

We suggest that alpine plant communities in Japan are vulnerable to climate change; however, the magnitude of decrease differs with respect to the different level of dependence of each community on topographic factors. Alpine plants in snowbed grassland and fellfield communities that depend less on topographic factors are considered to be more vulnerable to climate change.

### Sustainable and vulnerable populations of *L. muta japonica* in Japan

Our results showed that in the study area the majority (more than 99%) of potential habitat for *L. muta japonica* will disappear by 2081–2100, regardless of the uncertainty in GCMs (Figs. 3 and 6). A previous study that assessed the effects of climate change on *L. muta japonica* showed that 80% of *L. muta japonica* populations would disappear if the temperature rises by 3 °C [22]. In the Swiss Alps, Revermann et al. [18], using several SDMs. predicted a decrease of up to two-thirds in the potential habitat of *L. m. helvetica* by 2070, and a shift of potential habitat toward the mountain tops. Potential habitats for subspecies of *L. muta* throughout Europe were predicted to decrease progressively from approximately two million square kilometers at present to approximately one million square kilometers by 2080 [20]. In the 2080s, *L. muta japonica* populations are predicted to decrease even more sharply compared with European populations of *L. muta*, and thus may represent the *L. muta* subspecies most vulnerable to global warming. One reason for the high vulnerability of *L. muta japonica* is that the habitat in Japan is at the southern limit of the global distribution for this species [27]. A second reason is the difference in the altitude inhabited by *L. muta* species in the Swiss Alps compared to Japan’s northern Alps. The highest altitudinal peak in the study area is 3190 m, whereas the highest altitudinal peak in the Swiss Alps is 4609 m. In Japan, the alpine vegetation used by *L. muta japonica* already extends to the top and ridges of the mountains, whereas the nival zone, which is covered in snow throughout most of the year at present, occurs above the alpine zone in Europe. Therefore, in future, the alpine vegetation in Japan has little potential habitat above the current habitat range, whereas alpine vegetation in Europe has a larger potential habitat above the current alpine zone. Consequently, the negative influence of future climate change will be larger on *L. muta* habitats in Japan compared to those in Europe.

Sustainable potential habitats were predicted to occur in just three grid cells in the northwestern part of the study area under future climate conditions (Fig. 6a). Our results show that both climatic factors, including cooler temperature, greater snow, and lower summer precipitation, and topographic factors, such as closer to the ridge, leeward, and gentler slope, are positively important for sustaining the potential habitat of *L. muta japonica*. Potential habitats with GCM-related uncertainty were predicted to occur in the central and northwestern parts of the study area (Fig. 6b). Potential habitats should be considered high-priority candidates for investment in climate adaptation practices.

Some uncertainties may still remain regarding prediction of the future potential habitat of *L. muta japonica*. Plant species have plasticity to environmental change, which may moderate the decreasing speed of alpine plant community distributions. SDMs are based on the hypothesis that relationships between *L. muta japonica* and alpine plant communities will not change even after climate change (the hypothesis is called niche conservatism [53]). Variation among SDMs and greenhouse gas emission scenarios may also be a source of uncertainties regarding future predictions. However, we believe that these uncertainties would probably not change the general trends in our results, although detailed values may be changed.

## Conclusions and conservation planning for *L. muta japonica* based on habitat predictions

We showed that the potential habitats for *L. muta japonica* would dramatically decrease under different climate change scenarios, although limited potential habitats were predicted to be maintained. These sustainable potential habitats are considered as refugia for the survival of the population under future climatic conditions. Fortunately, all of these areas have already been included in protected areas; however, revision of the management policy, including raising the protection level, restricting human entry to the mountains, and tightening food waste management, are necessary for enhancing the protection of this species within these areas. Moreover, it is also necessary to monitor changes in the size of the *L. muta japonica* populations in the future. However, an increase in the numbers of predators (e.g., foxes) and grazing ungulates such as deer [54] may accelerate the decrease in *L. muta japonica* numbers before the effects of climate change become apparent.

Adaptive management, including preventing the invasion of competitive species, assisted breeding, and assisted migration, represents an alternative strategy to reduce the impact of climate change on species and their habitats. Ecological disturbance, such as an increase in competitors, may induce a marked decline in *L. muta japonica* populations, even in the sustainable habitats of this species. Shifts of terrestrial mammals (e.g., Japanese red fox *Vulpes vulpes japonica*, sika deer *C. nippon*, wild boar *Sus scrofa*, Japanese macaque *Macaca fuscata*, and Asian black bear *Ursus thibetanus*) into the alpine zone with changing climate may also accelerate the decline of *L. muta japonica*, due to an increase in predation pressure and competition for alpine plant species as food resources. The protection of chicks has already begun through the use of artificial cages, which reduce high mortality due to heavy rain in the early fledgling period and predation by terrestrial animals [55]. These types of adaptive management may be required to ensure the conservation of *L. muta japonica* under future climate conditions.

A nationwide-scale assessment of *L. muta japonica* populations is a future prospect. Nationwide-scale assessments are expected to help identify the most vulnerable populations and pinpoint candidate areas for assisted migration, as an alternative means of adaptive management. Assisted migration may be the only strategy to prevent the extinction of many montane species, particularly the alpine Rock Ptarmigan that was the focus of the present study, which face habitat loss as suitable climatic conditions migrate upward and off the top of mountain ranges [56]. For the planning of conservation strategies at a nationwide scale, we believe that our modeling approach, which describes the structure of the alpine ecosystems, is useful and could be applied to other regions.

## Additional files

**Additional file 1: Appendix S1.** The number of *L. muta japonica* territories and birds on the mountains in the study area.

**Additional file 2: Figure S1.** A map of alpine plant communities in the study area, which include Japanese stone pine (*Pinus pumila*) communities (shown in blue), snowbed grassland communities (green), and alpine fellfield communities (orange). The three plant communities were extracted from a digitized vegetation map provided by the Ministry of the Environment of Japan (http://www.biodic.go.jp/trialSystem/EN/info/vg67.html, accessed in September 2012).

**Additional file 3: Table S1.** Median and ranges (minimum to maximum) of four climatic variables under current (JMA 1996) and 24 future climate scenarios (Meehl et al. 2007) with their spatial resolutions in the study area (36.25–36.5°N, 137.5–137.7°E). WI: warmth index (Kira 1948), TMC: temperature of the coldest month, PRS: summer (May-September) precipitation, MSW: maximum snow water equivalent. Summary of the current climate was shown above the dashline, and summaries of 24 future climate scenarios were shown under the dashline.

**Additional file 4: Appendix S2.** The code for developing a generalized additive model (GAM) of sub-model A in R. Abbreviations in the code are as follows: data.csv in line 1, dataset which include response variable and explanation variables; RpPA in line 3, presence and absence data of territories of the rock ptarmigan; AfR in line 3, area fraction of alpine fellfield communities; SgR in line 3, area fraction of snowbed grassland communities; DistR in line 3, distance from the ridge; PpR in line 3, area fraction of *Pinus pumila* communities; STUDY_TIME in line 3, the number of surveys.

**Additional file 5: Table S2.** Akaike information criteria (AIC), differences of AIC from best model (ΔAIC) and Akaike weight for each model of sub-model A, which used a generalized additive model for predicting the distribution of *Lagopus muta japonica*. S(DR, AFPp) shows interaction smoothing term of distance from the ridge (DR) and area fraction of the *Pinus pumila* community (AFPp). S(AFFf) and s(AFSg) indicate the smoothing terms of area fraction of fellfield community and snowbed grassland community. Null means a null model without explanatory variables.

**Additional file 6: Figure S2.** The additive logistic fit of *Lagopus muta japonica* occurrence to four explanatory variables. Values on the y axis of (**A**) and (**B**), and contour lines in (**C**) show the linear predictor for the variables, which indicates the extent of positive and negative effects of each explanatory variable in the model. The dashed curves in (A) and (B) are pointwise two times of standard-error bands, which can be viewed as ~95% pointwise confidence intervals. Horizontal red dashed lines in (A) and (B) indicate the zero value of the estimate for each variable. Significant effects of explanatory variables on the model are shown in red, blue, and open boxes, which indicate positive, negative, and neutral, respectively, in (A) and (B). In (C), the warmer the color, the more positive the effect of the explanatory variables. Values on each contour line in (C) show the linear predictor for the two variables.

**Additional file 7: Table S3.** Akaike information criteria (AIC), differences of AIC from best model (∆AIC) and Akaike weight for each model of sub-model B1 which used a generalized additive model for predicting the distribution of alpine vegetation zone. S(WI, TMC) shows interaction smoothing term of warmth index (WI, Kira 1948) and minimum temperature of the coldest month (TMC). S(PRS) and s(MSW) indicate the smoothing terms of summer (May-September) precipitation and maximum snow water equivalent (MSW). Null means a null model without explanatory variables.

**Additional file 8: Figure S3.** The additive logistic fit of the alpine vegetation zone occurrence to four explanatory variables. Values on the y axis of (**A**) and (**B**), and contour lines in (**C**) show the linear predictor for the variables, which indicates the extent of positive and negative effects of each explanatory variable in the model. The dashed curves in (A) and (B) are pointwise two times of standard-error bands, which can be viewed as ~95% pointwise confidence intervals. Horizontal red dashed lines in (A) and (B) indicate the zero value of the estimate for each variable. Significant effects of explanatory variables on the model are shown in red, blue, and open boxes, which indicate positive, negative, and neutral, respectively, in (A) and (B). In (C), the warmer the color, the more positive the effect of the explanatory variables. Values on each contour line in (C) show the linear predictor for the two variables.

**Additional file 9: Figure S4.** Importance of climatic and topographic variables for three alpine plant communities in sub-model B2 (random forest). The importance of each variable was identified based on increased mean square errors (increased MSE; Breiman 2001).

## Data Availability

The datasets in this study are available from the corresponding author on reasonable request.

## Abbreviations

GCMs: general circulation models
SDMs: species distribution models
WCRP: World Climate Research Programme
CMIP3: Coupled Model Intercomparison Project phase 3
IPCC: Intergovernmental Panel on Climate Change
SAGA: System for Automated Geoscientific Analyses
TWI: topographic wetness index
GAM: generalized additive model
IOV: importance of the variables
AUC: area under the curve
ROC: receiver operating characteristic
RF: random forests
MSE: mean square errors
