# Supplementary material for: Modeling future wildlife habitat suitability: serious climate change impacts on the potential distribution of the Rock Ptarmigan Lagopus muta japonica in Japan’s northern Alps
Source: BMC Ecol. 2019 Jul 10;19:23. doi: 10.1186/s12898-019-0238-8 (PMC6617707; doi:10.1186/s12898-019-0238-8)
Supplement: Supplementary file 1 — Additional file 1: Appendix S1. The number of L. muta japonica territories and birds on the mountains in the study area. [file 12898_2019_238_MOESM1_ESM.docx]

**Additional file 1: Appendix 1.** The number of *L. muta japonica* territories and birds on the mountains in the study area.

| No | Mountains | Number of territories | Number of birds | Altitude of the top | Study years | References^1)^ |
| --- | --- | --- | --- | --- | --- | --- |
| 1 | Mt. Yakushi | 36 - 50 | 90 - 125 | 2,926 | 1974, 1993, 2010 | a, b, c |
| 2 | Mt. Taro | 2 - 4 | 5 - 10 | 2,373 | 1974, 1979, 1993, 2010 | a, b, c, d |
| 3 | Mt. Kitanomata, Mt. Akagi | 20 | 25 | 2,661, 2,622 | 1979 | d |
| 4 | Mt. Kurobegoro | 13 | 33 | 2,840 | 1978 | d |
| 5 | Mt. Mitsumatarenge | 13 | 33 | 2,841 | 1978 | d |
| 6 | Mt. Warimo, Mt. Washiba | 12 | 30 | 2,888, 2,924 | 1972 | e |
| 7 | Mt. Jii | 12 | 30 | 2,825 | 1985 | f |
| 8 | Kumonotaira plateau | 8 | 20 | 2,500 - 2,700 | 1985 | f |
| 9 | Mt. Suisho | 18 | 45 | 2,986 | 1988 | g |
| 10 | Mt. Akaushi | 27 | 68 | 2,864 | 1992 | h |
| 11 | Mt. Masago | 6 | 15 | 2,862 | 1972 | e |
| 12 | Mt. Noguchigoro | 16 | 40 | 2,924 | 1972 | e |
| 13 | Mt. Mitsu | 12 | 30 | 2,845 | 1972 | e |
| 14 | Mt. Eboshi | 1 | 3 | 2,628 | 1972 | e |
| 15 | Mt. Minamisawa | 1 | 3 | 2,625 | 1972 | e |
| 16 | Mt. Fudo | 2 | 5 | 2,601 | 1972 | e |
| 17 | Mt. Sugoroku | 19 | 48 | 2,860 | 2006 | i |
| 18 | Mt. Momisawa | 12 | 30 | 2,755 | 2006 | i |
| 19 | Mt. Kasa, Mt. Orido | 17 | 43 | 2,897, 2,813 | 1996 | j |
| 20 | Mt. Yari, Mt. Naka, Mt. Minami | 44 | 110 | 3,180, 3,084, 3,033 | 2006 | i |
| 21 | Hotaka Mountain Range | 35 | 88 | 3,190 | 2007 | i |
| 22 | Mt. Tsubakuro | 4 | 10 | 2,763 | 1971, 1972 | e |
| 23 | Mt. Otensho | 13 | 33 | 2,922 | 1979 | k |
| 24 | Mt. Jyonen | 3 - 4 | 8 - 10 | 2,857 | 1979, 2005 | i, k |
| 25 | Mt. Cho | 6 - 11 | 15 - 28 | 2,677 | 1979, 2005 | i, l |
| 26 | Mt. Otaki | 0 - 2 | 0 - 5 | 2,616 | 1979, 2005 | i, l |
| Total number | | 352 - 376 | 860 - 920 |  |  |  |

^1)^ The number of *L. muta japonica* territories on the mountains in the study area were quoted from the following literatures.

a: Toyama Pref. (1974) A brief of *Lagopus muta japonica* in Mt. Yakushi. (in Japanese)

b: Toyama Pref. (1993) A study of *Lagopus muta japonica* habitat in Mt. Yakushi, 1992. (in Japanese)

c: Society for the Study of Rock Ptarmigan in Toyama (2011) A study of *Lagopus muta japonica* status in the whole area of Mt. Yakushi, 2010. (in Japanese)

d: Toyama Pref. (1985) A study report of *Lagopus muta japonica* habitat in Mt. Kurobegoro, Mt. Kaminodake, Mt. Shirouma, Mt. Tateyama, Mt. Goryu and Mt. Karamatsu. (in Japanese)

e: Haneda, K. & Hirabayashi, K. (1974) Territory of Rock Ptarmigan (*Lagopus mutus japonicus)* in the backbone around the Takase River basin. A comprehensively investigative report of nature around the Takase River basin (ed. by the committee of the comprehensively investigation of nature around the Takase River basin), pp. 87-106. (in Japanese with English summary)

f: Toyama Pref. (1986) A study report of *Lagopus muta japonica* habitat in Kumonotaira plateau, 1985.

g: Society for the Study of Rock Ptarmigan in Toyama (1988) A study report of *Lagopus muta japonica* status in 1988. Toyama Pref. (in Japanese)

h: Toyama Pref. (1992) A study report of *Lagopus muta japonica* habitat in Mt. Akashi. (in Japanese)

i: CHUBU Regional Forest Office (2009) A study report of *Lagopus muta japonica* in 2004 - 2008. (in Japanese)

j: Gifu Pref., Wild Bird Society of Japan (1998) A second study report of *Lagopus muta japonica* in Mt. Norikura, Mt. Ontake and Mt. Kasagatake.

k: Haneda, K., Kamizima, T., Ota, H. and Nakamura, M. (1981) Estimation of territory distribution in Japanese Ptarmigans *Lagopus mutus japonicus* in Mt. Jonen and Mt. Otenjo. A comprehensively investigative report of nature and culture around the Karasu River, Japan's northern Alps. (ed. by the comprehensive scientific investigation group of the Karasu River, Japan’s northern Alps), pp. 349-359. (in Japanese with English summary)

l: Haneda, K., Furihata, K., Ota, H., Okubo, M., Nosaki, M. and Okuta, Y. (1981) Japanese Ptarmigans *Lagopus mutus japonicus* in Mt. Chogatake and Mt. Otaki. (ed. by the comprehensive scientific investigation group of the Karasu River, Japan’s northern Alps), pp. 361-374. (in Japanese with English summary)
