# Supplementary figures and images for: Modeling future wildlife habitat suitability: serious climate change impacts on the potential distribution of the Rock Ptarmigan Lagopus muta japonica in Japan’s northern Alps
Source: BMC Ecol. 2019 Jul 10;19:23. doi: 10.1186/s12898-019-0238-8 (PMC6617707; doi:10.1186/s12898-019-0238-8)

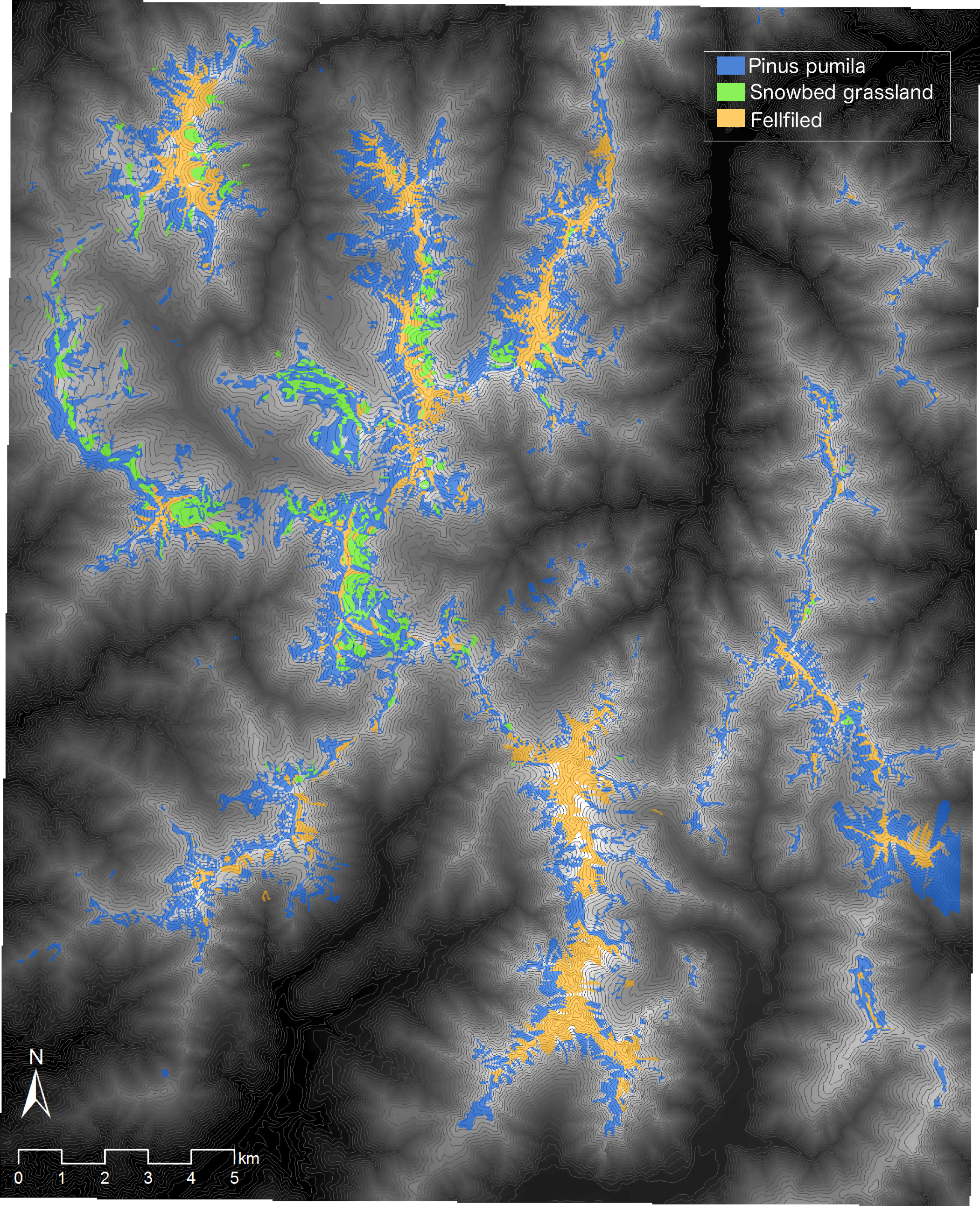

Supplement: Supplementary file 2 — Additional file 2: Figure S1. A map of alpine plant communities in the study area, which include Japanese stone pine (Pinus pumila) communities (shown in blue), snowbed grassland communities (green), and alpine fellfield communities (orange). The three plant communities were extracted from a digitized vegetation map provided by the Ministry of the Environment of Japan (http://www.biodic.go.jp/trialSystem/EN/info/vg67.html, accessed in September 2012). [file 12898_2019_238_MOESM2_ESM.tiff]

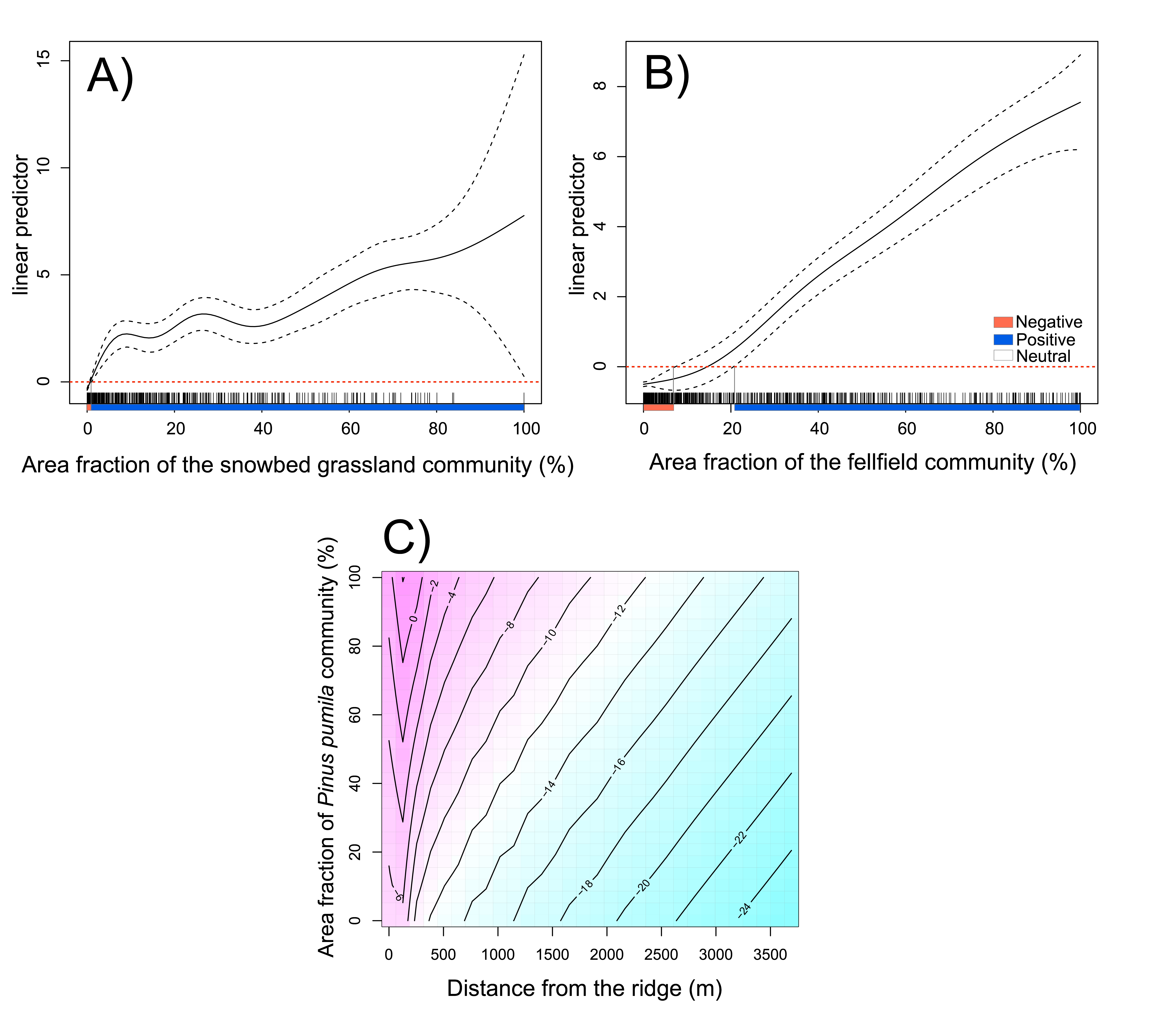

Supplement: Supplementary file 6 — Additional file 6: Figure S2. The additive logistic fit of Lagopus muta japonica occurrence to four explanatory variables. Values on the y axis of (A) and (B), and contour lines in (C) show the linear predictor for the variables, which indicates the extent of positive and negative effects of each explanatory variable in the model. The dashed curves in (A) and (B) are pointwise two times of standard-error bands, which can be viewed as ~95% pointwise confidence intervals. Horizontal red dashed lines in (A) and (B) indicate the zero value of the estimate for each variable. Significant effects of explanatory variables on the model are shown in red, blue, and open boxes, which indicate positive, negative, and neutral, respectively, in (A) and (B). In (C), the warmer the color, the more positive the effect of the explanatory variables. Values on each contour line in (C) show the linear predictor for the two variables. [file 12898_2019_238_MOESM6_ESM.tiff]

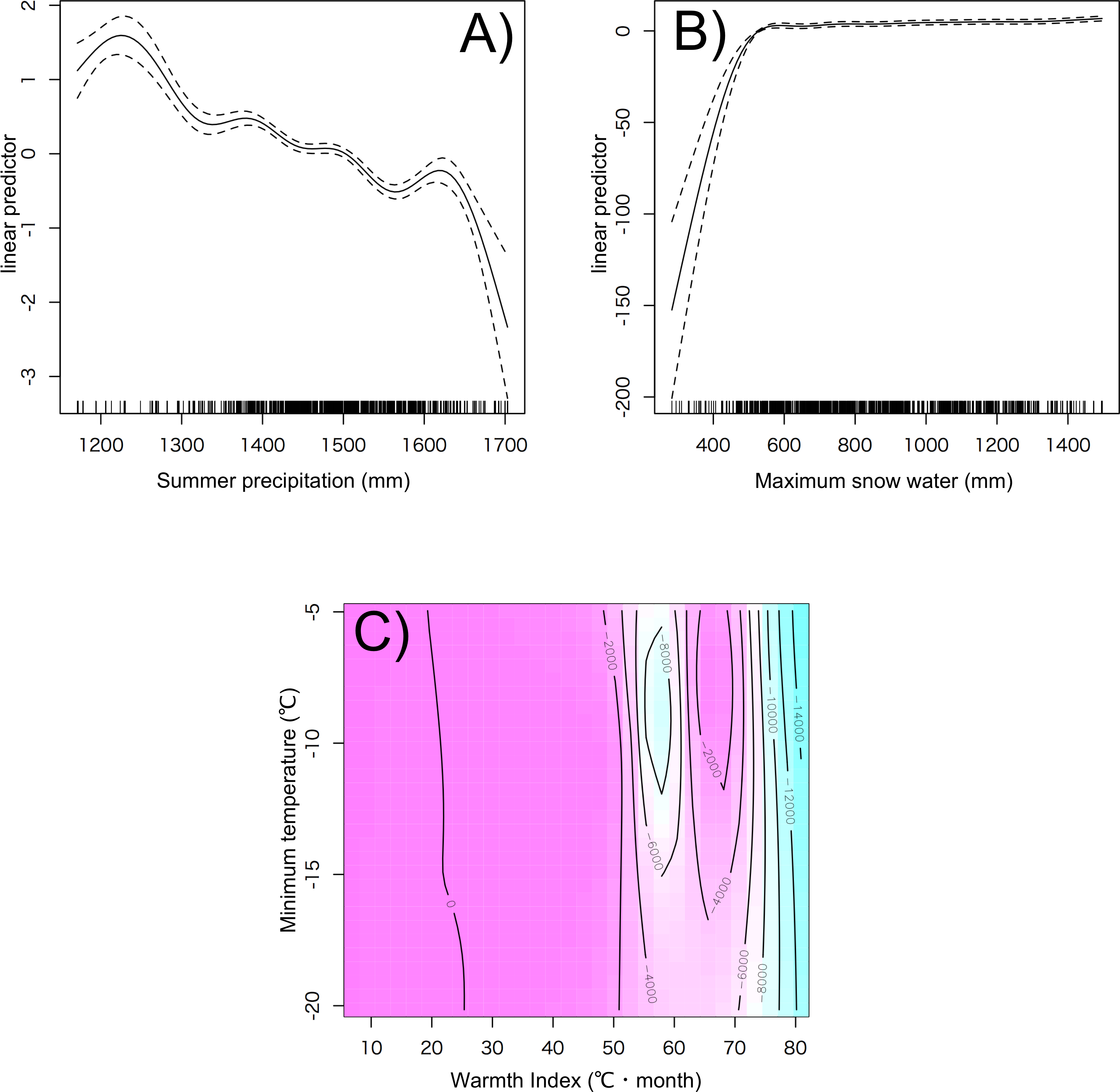

Supplement: Supplementary file 8 — Additional file 8: Figure S3. The additive logistic fit of the alpine vegetation zone occurrence to four explanatory variables. Values on the y axis of (A) and (B), and contour lines in (C) show the linear predictor for the variables, which indicates the extent of positive and negative effects of each explanatory variable in the model. The dashed curves in (A) and (B) are pointwise two times of standard-error bands, which can be viewed as ~95% pointwise confidence intervals. Horizontal red dashed lines in (A) and (B) indicate the zero value of the estimate for each variable. Significant effects of explanatory variables on the model are shown in red, blue, and open boxes, which indicate positive, negative, and neutral, respectively, in (A) and (B). In (C), the warmer the color, the more positive the effect of the explanatory variables. Values on each contour line in (C) show the linear predictor for the two variables. [file 12898_2019_238_MOESM8_ESM.tiff]

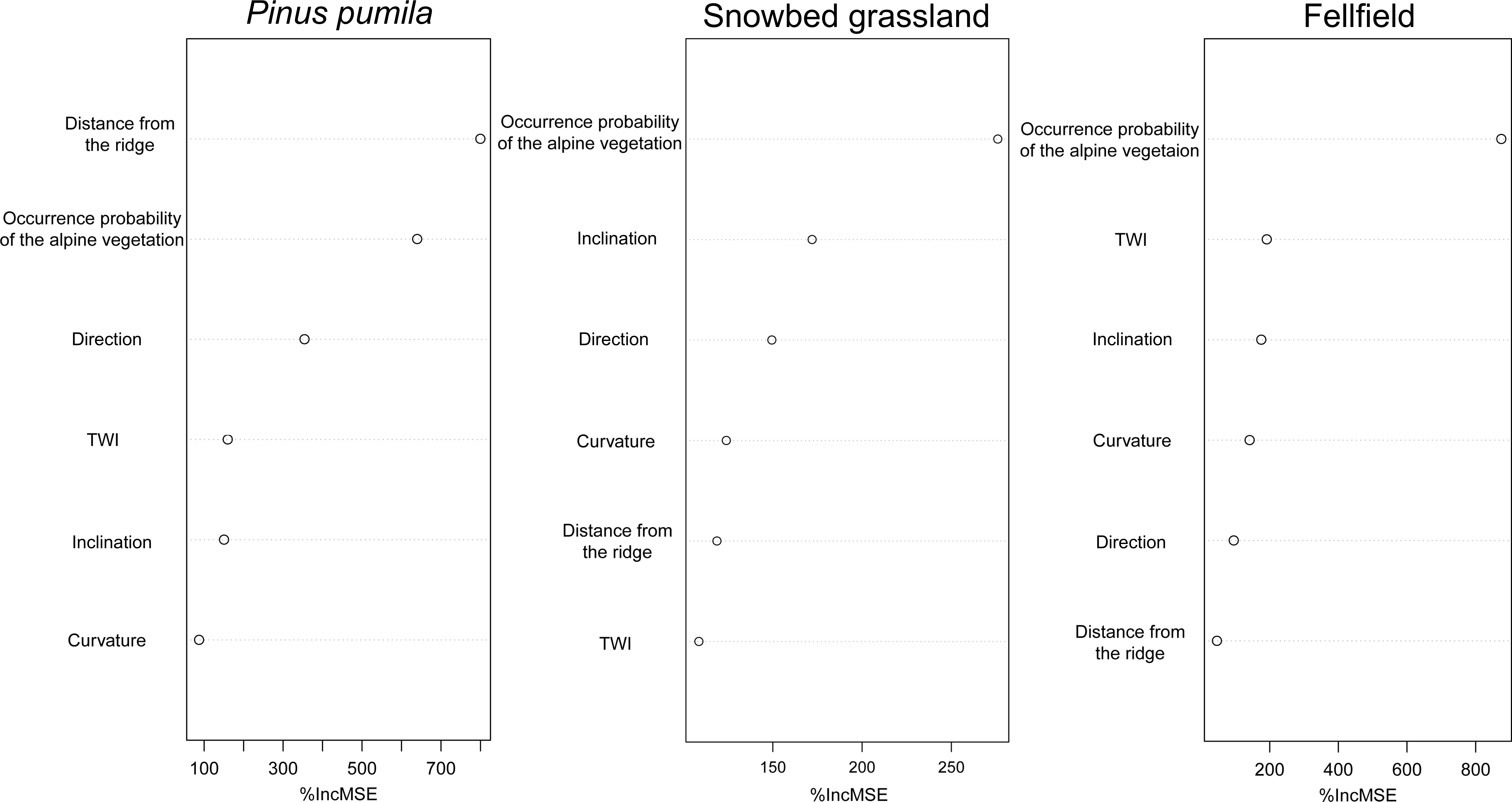

Supplement: Supplementary file 9 — Additional file 9: Figure S4. Importance of climatic and topographic variables for three alpine plant communities in sub-model B2 (random forest). The importance of each variable was identified based on increased mean square errors (increased MSE; Breiman 2001). [file 12898_2019_238_MOESM9_ESM.tiff]
