# Supplementary material for: Modeling future wildlife habitat suitability: serious climate change impacts on the potential distribution of the Rock Ptarmigan Lagopus muta japonica in Japan’s northern Alps
Source: BMC Ecol. 2019 Jul 10;19:23. doi: 10.1186/s12898-019-0238-8 (PMC6617707; doi:10.1186/s12898-019-0238-8)
Supplement: Supplementary file 3 — Additional file 3: Table S1. Median and ranges (minimum to maximum) of four climatic variables under current (JMA 1996) and 24 future climate scenarios (Meehl et al. 2007) with their spatial resolutions in the study area (36.25–36.5°N, 137.5–137.7°E). WI: warmth index (Kira 1948), TMC: temperature of the coldest month, PRS: summer (May-September) precipitation, MSW: maximum snow water equivalent. Summary of the current climate was shown above the dashline, and summaries of 24 future climate scenarios were shown under the dashline. [file 12898_2019_238_MOESM3_ESM.docx]

**Additional file 3: Table S1.** Median and ranges (minimum to maximum) of four climatic variables under current (JMA 1996) and 24 future climate scenarios (Meehl et al. 2007) with their spatial resolutions in the study area (36.25–36.5°N, 137.5–137.7°E). WI: warmth index (Kira 1948), TMC: temperature of the coldest month, PRS: summer (May-September) precipitation, MSW: maximum snow water equivalent. Summary of the current climate was shown above the dashline, and summaries of 24 future climate scenarios were shown under the dashline.

| ID number | Climatic data | Spatial resolution  (degree) | WI (˚C·month) | TMC (˚C) | PRS (mm) | MSW (mm) |
| --- | --- | --- | --- | --- | --- | --- |
| - | Current | 0.0083N × 0.0125E | 34.3 (6.8–80.9) | −13.5 (-20.2–−5.0) | 1482 (1171–1703) | 828 (283–1496) |
| 1 | BCCR-BCM2.0 | 2.8125N × 2.8125E | 49.8 (18.6–101.1) | −11.3 (-18.0–−2.6) | 1707 (1347–1969) | 741 (155–1537) |
| 2 | CCSM3 | 1.4063N × 1.4063E | 50.3 (18.1–102.1) | −11.3 (-18.1–−2.3) | 1756 (1384–2030) | 655 (140–1336) |
| 3 | CGCM3.1(T47) | 3.7500N × 3.7500E | 52.9 (21.0–105.1) | −10.1 (-16.5–−1.8) | 1771 (1390–2051) | 738 (131–1577) |
| 4 | CGCM3.1(T63) | 2.8125N × 2.8125E | 52.0 (20.4–104.0) | −10.6 (-17.2–−2.2) | 1711 (1340–1975) | 686 (132–1474) |
| 5 | CNRM-CM3 | 2.8125N × 2.8125E | 54.6 (22.0–106.7) | −10.1 (-16.3–−1.8) | 1574 (1239–1811) | 656 (83–1474) |
| 6 | CSIRO-Mk3.0 | 1.8750N × 1.8750E | 45.4 (15.3–96.5) | −11.7 (−18.3–−3.3) | 1510 (1180–1745) | 713 (181–1482) |
| 7 | CSIRO-Mk3.5 | 1.8750N × 1.8750E | 52.9 (20.2–105.0) | −10.0 (−16.7–−1.4) | 1493 (1164–1723) | 465 (56–1053) |
| 8 | ECHAM5/MPI-OM | 1.8750N × 1.8750E | 53.1 (20.3–106.4) | −9.5 (−16.2–−0.77) | 1156 (396–3847) | 651 (87–1453) |
| 9 | ECHO-G | 3.7500N × 3.7500E | 59.9 (26.6–113.6) | *−*9.1 (*−*15.7–*−*0.65) | 1638 (1280–1897) | 609 (69–1383) |
| 10 | FGOALS-g1.0 | 2.8125N × 2.8125E | 46.6 (15.9–97.3) | −11.2 (−17.8–−2.7) | 1672 (1315–1935) | 689 (143–1403) |
| 11 | GFDL-CM2.0 | 2.5000N × 2.0000E | 53.7 (20.9–106.9) | −9.4 (−16.0–−0.96) | 1828 (1415–2114) | 642 (79–1432) |
| 12 | GFDL-CM2.1 | 2.5000N × 2.0000E | 53.6 (20.9–106.0) | −8.8 (−15.4–−0.4) | 1571 (1240–1807) | 532 (48–1226) |
| 13 | GISS-AOM | 4.0000N × 3.0000E | 47.2 (15.8–98.8) | −11.2 (−17.7–−2.8) | 1416 (1112–1626) | 741 (157–1564) |
| 14 | GISS-EH | 5.0000N × 3.9130E | 48.9 (17.4–101.0) | −8.8 (−15.1–−0.46) | 1709 (1348–1971) | 656 (65–1503) |
| 15 | GISS-ER | 5.0000N × 3.9130E | 49.7 (17.7–102.4) | −10.7 (−17.2–−2.3) | 350 (278–401) | 148 (23–325) |
| 16 | INGV-SXG | 1.1250N × 1.1250E | 50.7 (19.1–102.1) | −10.4 (−16.9–−2.0) | 1500 (1184–1730) | 695 (117–1447) |
| 17 | INM-CM3.0 | 5.0000N × 4.0000E | 51.0 (18.4–103.5) | −10.5 (−16.7–−2.3) | 1452 (1125–1679) | 537 (96–1145) |
| 18 | IPSL-CM4 | 3.7500N × 2.5000E | 60.0 (26.5–113.7) | −9.5 (−16.2–−0.66) | 1543 (1203–1776) | 629 (76–1364) |
| 19 | MIROC3.2(hires) | 1.1250N × 1.1250E | 63.2 (29.0–117.8) | −8.4 (−15.1–0.25) | 1761 (1377–2032) | 495 (46–1148) |
| 20 | MIROC3.2(medres) | 2.8125N × 2.8125E | 57.7 (24.5–111.2) | −9.3 (−15.7–−0.88) | 1797 (1425–2072) | 707 (93–1535) |
| 21 | MRI-CGCM2.3.2 | 2.8125N × 2.8125E | 50.2 (18.4–101.2) | −10.9 (−17.3–−2.6) | 1640 (1304–1886) | 683 (119–1408) |
| 22 | PCM | 2.8125N × 2.8125E | 46.6 (15.8–97.2) | −10.9 (−17.1–−2.5) | 1517 (1176–1748) | 661 (135–1348) |
| 23 | UKMO-HadCM3 | 3.7500N × 2.5000E | 65.6 (31.7–120.0) | −8.5 (−14.9–−0.17) | 1740 (1381–2004) | 604 (57–1404) |
| 24 | UKMO-HadGEM1 | 1.8750N × 1.2500E | 60.3 (27.2–113.7) | −9.6 (−15.8–−1.3) | 1478 (1174–1692) | 662 (96–1489) |
