# Supplementary material for: Modeling future wildlife habitat suitability: serious climate change impacts on the potential distribution of the Rock Ptarmigan Lagopus muta japonica in Japan’s northern Alps
Source: BMC Ecol. 2019 Jul 10;19:23. doi: 10.1186/s12898-019-0238-8 (PMC6617707; doi:10.1186/s12898-019-0238-8)
Supplement: Supplementary file 4 — Additional file 4: Appendix S2. The code for developing a generalized additive model (GAM) of sub-model A in R. Abbreviations in the code are as follows: data.csv in line 1, dataset which include response variable and explanation variables; RpPA in line 3, presence and absence data of territories of the rock ptarmigan; AfR in line 3, area fraction of alpine fellfield communities; SgR in line 3, area fraction of snowbed grassland communities; DistR in line 3, distance from the ridge; PpR in line 3, area fraction of Pinus pumila communities; STUDY_TIME in line 3, the number of surveys. [file 12898_2019_238_MOESM4_ESM.docx]

**Additional file 4: Appendix S2.** The code for developing a generalized additive model (GAM) of sub-model A in R. Abbreviations in the code are as follows: data.csv in line 1, dataset which include response variable and explanation variables; RpPA in line 3, presence and absence data of territories of the rock ptarmigan; AfR in line 3, area fraction of alpine fellfield communities; SgR in line 3, area fraction of snowbed grassland communities; DistR in line 3, distance from the ridge; PpR in line 3, area fraction of *Pinus pumila* communities; STUDY_TIME in line 3, the number of surveys.

d<- read.table("data.csv", sep=",", header=TRUE)

library(mgcv)

gamRp <- gam(d$RpPA~s(AfR)+s(SgR)+s(DistR, PpR), family=binomial, data=d, maxit=1000, offset=d$STUDY_TIME)
