# Supplementary material for: Modeling future wildlife habitat suitability: serious climate change impacts on the potential distribution of the Rock Ptarmigan Lagopus muta japonica in Japan’s northern Alps
Source: BMC Ecol. 2019 Jul 10;19:23. doi: 10.1186/s12898-019-0238-8 (PMC6617707; doi:10.1186/s12898-019-0238-8)
Supplement: Supplementary file 5 — Additional file 5: Table S2. Akaike information criteria (AIC), differences of AIC from best model (ΔAIC) and Akaike weight for each model of sub-model A, which used a generalized additive model for predicting the distribution of Lagopus muta japonica. S(DR, AFPp) shows interaction smoothing term of distance from the ridge (DR) and area fraction of the Pinus pumila community (AFPp). S(AFFf) and s(AFSg) indicate the smoothing terms of area fraction of fellfield community and snowbed grassland community. Null means a null model without explanatory variables. [file 12898_2019_238_MOESM5_ESM.docx]

**Additional File 5: Table S2.** Akaike information criteria (AIC), differences of AIC from best model (ΔAIC) and Akaike weight for each model of sub-model A, which used a generalized additive model for predicting the distribution of *Lagopus muta japonica*. S(DR, AFPp) shows interaction smoothing term of distance from the ridge (DR) and area fraction of the *Pinus pumila* community (AFPp). S(AFFf) and s(AFSg) indicate the smoothing terms of area fraction of fellfield community and snowbed grassland community. Null means a null model without explanatory variables.

| Models | AIC | ΔAIC | Akaike weight |
| --- | --- | --- | --- |
| s(DR, AFPp)+s(AFFf)+s(AFSg) | 882.4 | 0.00 | 1.0 |
| s(DR, AFPp)+s(AFFf) | 1242.6 | 360.16 | 0.0 |
| s(AFFf)+s(AFSg) | 1303.3 | 420.84 | 0.0 |
| s(DR, AFPp)+s(AFSg) | 1578.8 | 696.34 | 0.0 |
| s(AFFf) | 1807.2 | 924.79 | 0.0 |
| s(DR, AFPp) | 1823.8 | 941.34 | 0.0 |
| s(AFSg) | 2109.5 | 1227.2 | 0.0 |
| Null | 2628.7 | 1746.3 | 0.0 |
