# Supplementary material for: Modeling future wildlife habitat suitability: serious climate change impacts on the potential distribution of the Rock Ptarmigan Lagopus muta japonica in Japan’s northern Alps
Source: BMC Ecol. 2019 Jul 10;19:23. doi: 10.1186/s12898-019-0238-8 (PMC6617707; doi:10.1186/s12898-019-0238-8)
Supplement: Supplementary file 7 — Additional file 7: Table S3. Akaike information criteria (AIC), differences of AIC from best model (∆AIC) and Akaike weight for each model of sub-model B1 which used a generalized additive model for predicting the distribution of alpine vegetation zone. S(WI, TMC) shows interaction smoothing term of warmth index (WI, Kira 1948) and minimum temperature of the coldest month (TMC). S(PRS) and s(MSW) indicate the smoothing terms of summer (May-September) precipitation and maximum snow water equivalent (MSW). Null means a null model without explanatory variables. [file 12898_2019_238_MOESM7_ESM.docx]

**Additional File 7: Table S3.** Akaike information criteria (AIC), differences of AIC from best model (∆AIC) and Akaike weight for each model of sub-model B1 which used a generalized additive model for predicting the distribution of alpine vegetation zone. S(WI, TMC) shows interaction smoothing term of warmth index (WI, Kira 1948) and minimum temperature of the coldest month (TMC). S(PRS) and s(MSW) indicate the smoothing terms of summer (May-September) precipitation and maximum snow water equivalent (MSW). Null means a null model without explanatory variables.

| Models | AIC | ∆AIC | Akaike weight |
| --- | --- | --- | --- |
| s(WI, TMC)+s(PRS)+s(MSW) | 24974.5 | 0.00 | 1.0 |
| s(WI, TMC)+s(MSW) | 25387.6 | 413.08 | 0.0 |
| s(WI, TMC)+s(PRS) | 26495.0 | 1520.45 | 0.0 |
| s(WI, TMC) | 26975.1 | 2000.61 | 0.0 |
| s(PRS)+s(MSW) | 46083.3 | 21108.75 | 0.0 |
| s(MSW) | 49503.4 | 24528.93 | 0.0 |
| s(PRS) | 57698.0 | 32723.51 | 0.0 |
| Null | 61362.7 | 36388.21 | 0.0 |
